# When Data Augmentation Falls Short: Wi-Fi Fingerprint-Based Indoor Localization Revisited

**DOI:** 10.3390/s26175392

**Published:** 2026-08-26

**Authors:** Nurbek Malikov, Marko Ristin, Shinnazar Seytnazarov

**Affiliations:** 1 School of Computing and Artificial Intelligence (SCAI), Nazarbayev University, Astana 010000, Kazakhstan; nurbek.malikov@nu.edu.kz; 2 Institute of Mechatronic Systems (IMS), Zurich University of Applied Sciences, 8401 Winterthur, Switzerland; rist@zhaw.ch

**Keywords:** indoor localization, Wi-Fi fingerprinting, RSSI, generative data augmentation, diffusion model, spatial sparsity

## Abstract

Generative data augmentation has been widely explored in Wi-Fi fingerprint-based indoor localization to reduce the cost of dense radiomap construction, with many studies reporting substantial localization improvements. However, these comparisons typically rely on default or weakly optimized baseline regressors, making it difficult to determine whether reported gains reflect genuine synthesis quality or merely compensate for suboptimal baselines. In this paper, we systematically investigate under which conditions generative augmentation is actually justified. We fine-tune four widely used localization regressors—kNN, SVR, XGBoost, and DNN—using Bayesian hyperparameter optimization and establish strong non-augmented baselines across radiomaps with controlled levels of spatial sparsity, constructed via farthest-point sampling. We then train five representative generative models—VAE, GAN, DDPM, DiT, and TDPM—within a unified augmentation pipeline that includes quality filtering and pseudo-labeling, and benchmark them against these baselines. Using two publicly available datasets, we show that none of the generative models consistently outperforms a non-augmented, fine-tuned baseline regressor such as XGBoost or kNN, across a wide range of sparsity levels. We further show that these conclusions are robust to three potential confounders: various proportions of synthetic data, the choice of localization regressor (ruling out circularity with the pseudo-labeling model), and the dataset itself, since the findings on the first dataset replicate on a second, structurally different building. These findings suggest that reported augmentation benefits in prior work may partly reflect under-optimized baselines rather than genuine synthesis quality, and that generative augmentation should be treated as a conditional last resort rather than a universal improvement strategy.

## 1. Introduction

Wi-Fi Received Signal Strength Indicator (RSSI) fingerprinting is one of the most widely studied and deployed techniques for indoor localization because it leverages existing Wi-Fi infrastructure without requiring additional hardware [1,2]. In a typical fingerprinting system, RSSI measurements are collected at a set of reference points (RPs) during an offline survey phase and stored in a radiomap. During the online application phase, a user device measures a new RSSI fingerprint, and a localization model (e.g., regressors such as k-nearest neighbors (kNN) or Deep Neural Networks (DNNs)) estimates the user position by matching the observed fingerprint against the radiomap.

The localization accuracy of fingerprint-based systems strongly depends on radiomap density and coverage. In general, denser radiomaps provide better positioning accuracy because the localization model can learn finer spatial variations in RSSI patterns. However, constructing dense radiomaps is labor-intensive and expensive, as fingerprints must be collected manually throughout the environment. Furthermore, environmental changes such as furniture relocation, human activity, or modifications to the Wi-Fi infrastructure gradually reduce radiomap quality, requiring periodic maintenance and re-surveying [1,2,3,4].

To alleviate the cost of radiomap construction, recent studies have explored the use of generative models for fingerprint synthesis and data augmentation. Popular approaches employ variational autoencoders (VAEs) [5,6], Generative Adversarial Networks (GANs) [7,8,9], and, more recently, diffusion-based models [10,11,12]. The common idea is to generate synthetic RSSI fingerprints and incorporate them into the radiomap, thereby increasing the amount of training data available to the localization model. Several studies report substantial reductions in localization error, particularly when only a fraction of the original radiomap is available (see Section 2.3).

Despite these promising results, an important question remains largely unexplored. Existing studies typically compare the performance of localization models trained on augmented radiomaps against baseline localization models that are trained on sparse radiomaps, which include only real fingerprints. However, the baseline localization models are often trained with default configurations, and details regarding hyperparameter optimization are frequently omitted. Consequently, it is difficult to determine whether the reported improvements originate from the synthetic fingerprints themselves or from the fact that augmentation compensates for suboptimal baseline models.

This distinction is important from both scientific and practical perspectives. Training modern generative models can require substantial computational resources, specialized hardware, and considerable engineering effort. In contrast, localization regressors such as XGBoost, kNN, Support Vector Regression (SVR), and DNNs can often be optimized using relatively inexpensive hyperparameter search procedures. If careful optimization of baseline regressors yields performance comparable to that of augmented pipelines, then the practical value of generative augmentation may be significantly smaller than suggested by the existing literature.

In this paper, we revisit the role of generative augmentation in Wi-Fi fingerprint-based indoor localization. Rather than asking whether synthetic fingerprints can improve localization accuracy, we investigate under which conditions such augmentation is actually justified. To answer this question, we first construct radiomaps with controlled levels of spatial sparsity using a farthest-point sampling strategy. We then optimize four widely used localization regressors using Bayesian hyperparameter optimization and establish strong non-augmented baselines across a range of sparsity levels. Next, we evaluate five representative generative models—VAE, GAN, DDPM, diffusion transformer (DiT), and tabular diffusion probabilistic model (TDPM)—within a unified augmentation pipeline that includes quality filtering, pseudo-labeling, and downstream localization evaluation. Beyond this core comparison, we report five additional analyses: (i) an evaluation of the impact of various synthetic fingerprint proportions on localization accuracy, establishing that our conclusions are not an artifact of a single fixed synthesis volume; (ii) a repeated-seed ablation of the quality-filtering pipeline; (iii) a circularity check that varies the downstream localization regressor independently of the pseudo-labeling regressor; (iv) an evaluation of a genuinely coordinate-conditional generator that needs no pseudo-labels at all; and (v) a replication of the entire study on a second dataset from a structurally different building.

Our results reveal that none of the evaluated generative models consistently outperforms a properly tuned non-augmented baseline regressor such as XGBoost or kNN across various sparsity levels. These findings suggest that some of the improvements reported in the literature may be attributable to weak baseline configurations rather than to augmentation itself.

The remainder of the paper is organized as follows. Section 2 reviews the background and related work. Section 3 presents the methodology and augmentation pipeline. Section 4 discusses the experimental results. Finally, Section 5 concludes the paper and outlines future research directions.

## 2. Background and Related Work

### 2.1. Wi-Fi RSSI Fingerprinting

Wi-Fi fingerprinting relies on the concept of a radiomap. A radiomap

(1) $$R={\left\{\left(r_{i},p_{i}\right)\right\}}_{i=1}^{N_{s}}$$

consists of $N_{s}$ radiomap entries, where $r_{i}\in R^{D}$ denotes an RSSI fingerprint and $p_{i}=\left(x_{i},y_{i}\right)\in R^{2}$ denotes the coordinates of the reference point (RP) at which the fingerprint was collected.

A *reference point (RP)* is a physical survey location, while a *fingerprint* is an RSSI vector measured at that location. Multiple fingerprints may be collected at the same RP to capture temporal RSSI variations. Therefore, the number of radiomap entries $N_{s}$ may be larger than the number of unique RPs, denoted by $N_{RP}$.

During the online phase, a user device measures a query fingerprint $r_{q}$, and the localization system estimates its position

(2) $${\hat{p}}_{q}=f\left(r_{q}\right),$$

where $f:R^{D}\to R^{2}$ is the localization function learned from the radiomap. The goal of fingerprint-based indoor localization is to accurately approximate this mapping from RSSI fingerprints to spatial coordinates [1,2,13].

Localization performance depends heavily on the quality, quantity, and spatial distribution of the radiomap entries. In particular, a reduction in the number of surveyed RPs decreases the spatial coverage of the environment, whereas a reduction in the number of fingerprints collected at each RP limits the ability to model RSSI variability. Consequently, both RP density and fingerprint diversity influence localization accuracy.

### 2.2. Regression Models for Localization

The mapping from RSSI fingerprints to spatial coordinates has been widely studied using regression-based localization models [1,2,14,15]. In this paper, we consider four representative regression families: k-nearest neighbors (kNN), Support Vector Regression (SVR), eXtreme Gradient Boosting (XGBoost), and Deep Neural Networks (DNNs). These models were selected because they cover major classes of fingerprint-based localization methods: instance-based learning, kernel methods, tree-based ensemble learning, and deep learning.

Evaluating multiple regression families is important for two reasons. First, the best localization model may depend on radiomap density. For example, instance-based methods may behave differently from tree-based or neural models when the radiomap becomes sparse. Second, the apparent benefit of synthetic augmentation may depend on the strength of the downstream regressor. If the baseline regressor is weak or poorly tuned, augmentation may appear beneficial even when similar gains could be achieved through model optimization alone.

kNN is one of the earliest and most widely used baselines for Wi-Fi fingerprinting. Given a query fingerprint, kNN retrieves the *k* most similar fingerprints from the radiomap and estimates the position using the coordinates of the selected neighbors, often through an average or distance-weighted average. The method is simple, non-parametric, and easy to implement [16]. However, its accuracy depends strongly on radiomap density and spatial coverage. When nearby fingerprints are unavailable, kNN may select distant neighbors and produce large localization errors.

SVR extends Support Vector Machines to regression. It learns a function that predicts spatial coordinates from RSSI fingerprints while optimizing the margin between the predicted function and the training samples, thereby balancing prediction accuracy and model complexity through regularization [17]. SVR can be effective for nonlinear regression when suitable kernels and hyperparameters are selected. However, its performance is sensitive to hyperparameter choices, and training can become computationally expensive as the number of training samples increases. In high-dimensional RSSI spaces, SVR may also require careful preprocessing or feature selection.

XGBoost is a scalable gradient-boosted decision-tree ensemble. It builds an additive model by sequentially training decision trees to reduce prediction errors. XGBoost is particularly effective on tabular data and includes regularization mechanisms that help reduce overfitting [18]. These characteristics make it suitable for RSSI fingerprinting, where each fingerprint can be interpreted as a tabular feature vector and some AP observations may be missing or weak. Because of its strong empirical performance on structured data, XGBoost represents a strong baseline against which the additional value of synthetic augmentation can be evaluated.

DNNs learn complex nonlinear mappings by passing input data through multiple layers of interconnected neurons, each computing a weighted sum of its inputs followed by a nonlinear activation function to extract progressively higher-level representations. In indoor localization, DNNs can model complex relationships between AP signal patterns and spatial coordinates. Prior studies have explored multilayer perceptrons [14], convolutional NNs [19,20], and recurrent NNs [21] for fingerprint-based localization. However, DNNs are often data-hungry and sensitive to architecture, optimization settings, and regularization. In sparse radiomap settings, they may overfit unless carefully tuned [15].

In this work, these four regressors are not used merely as default baselines. Instead, their hyperparameters are systematically optimized to establish strong non-augmented references. This is essential for evaluating whether synthetic fingerprints provide genuine improvements beyond what can be achieved by careful baseline optimization.

### 2.3. Generative Models for Fingerprint Augmentation

Generative models aim to learn the underlying distribution of the training data and generate new samples that resemble real observations [5,10,22]. In Wi-Fi fingerprint-based localization, generative models are used to synthesize RSSI fingerprints with the goal of enlarging the training set and improving localization accuracy. After generation, coordinates are predicted for synthetic fingerprints, which are then added to the radiomap as synthetic entries to help regularize the regressor and reduce overfitting [23].

Generative augmentation methods differ in how they model the fingerprint distribution. Earlier approaches commonly rely on latent-variable models such as Variational Autoencoders (VAEs) or adversarial learning frameworks such as Generative Adversarial Networks (GANs). More recent approaches employ diffusion-based models, which generate data through iterative denoising. These model families differ in terms of training stability, sample diversity, computational cost, and suitability for sparse RSSI data.

#### 2.3.1. Variational Autoencoders (VAEs)

Variational Autoencoders (VAEs) are latent-variable generative models that combine neural networks with probabilistic inference. A VAE consists of an encoder that maps an input sample to a latent distribution and a decoder that reconstructs the sample from a latent variable. The model is trained by balancing reconstruction accuracy with a regularization term that encourages the latent space to follow a prior distribution, typically a Gaussian distribution [5].

For Wi-Fi fingerprint augmentation, VAEs can learn compact latent representations of RSSI fingerprints and generate new samples by decoding latent vectors [6,24,25,26,27]. Their training is generally stable compared to adversarial models. However, VAEs often produce over-smoothed samples because of the trade-off between reconstruction and regularization. This can be problematic for RSSI fingerprints, which may contain sparse, sharp, and location-dependent signal patterns.

#### 2.3.2. Generative Adversarial Networks (GANs)

Generative Adversarial Networks (GANs) formulate data generation as a game between two neural networks: a generator and a discriminator. The generator produces synthetic samples, while the discriminator attempts to distinguish generated samples from real ones. Through this adversarial training process, the generator learns to produce samples that resemble the training distribution [22].

GANs have been widely used for data augmentation because they can generate sharp and realistic samples. In Wi-Fi fingerprinting, GAN-based methods have been used to synthesize missing fingerprints or inpaint incomplete radiomaps [8,9,28,29,30]. Some studies have reportedly demonstrated positioning accuracy gains ranging from 15% to 37.51% in scenarios where half of the training data was generated using GANs [8,9]. However, GAN training can be unstable and may suffer from mode collapse, where the generator produces a limited variety of samples. These issues are particularly relevant in RSSI fingerprinting because the available training data are often limited and spatially sparse.

#### 2.3.3. Denoising Diffusion Probabilistic Models (DDPMs)

Denoising Diffusion Probabilistic Models (DDPMs) generate samples through a gradual denoising process. During training, noise is progressively added to real samples, and the model learns to reverse this process by predicting and removing noise. During generation, the model starts from random noise and iteratively denoises it until a realistic sample is obtained [10].

Diffusion models are attractive for fingerprint synthesis because they provide stable training and can model complex data distributions [11,12,31,32,33]. Compared to GANs, they are less prone to adversarial training instability and mode collapse. However, generation is computationally expensive because it typically requires many denoising steps. For RSSI fingerprint augmentation, this cost must be justified by measurable improvements in downstream localization accuracy.

To better accommodate tabular fingerprint data, specialized diffusion variants have been proposed. For example, a study in [12] adapts diffusion-style denoising to tabular RSSI settings. Conditioning on spatial coordinates turns the model into a conditional diffusion model, allowing it to generate fingerprints that are consistent with a target location.

#### 2.3.4. Diffusion Transformers (DiTs)

Diffusion Transformers (DiTs) combine diffusion-based generation with transformer architectures. Instead of using only multilayer perceptrons or convolutional architectures as denoising networks, diffusion transformers employ self-attention mechanisms to model dependencies among input features [34]. For RSSI fingerprints, this can be interpreted as modeling relationships among AP signal values [35].

The main advantage of diffusion transformers is their representational capacity. They can capture complex dependencies across features and have shown strong performance in modern generative modeling tasks. However, they are computationally intensive and may require substantial training resources. In the context of indoor localization, their practical value depends on whether the resulting synthetic fingerprints provide localization gains that justify this added complexity.

### 2.4. Limitations of Existing Augmentation Studies

Although many studies report localization improvements through synthetic fingerprint generation, the practical conditions under which augmentation is truly beneficial remain insufficiently understood [6,8,9,11,12,24,25,26,27,28,29,30,31,32,33,35].

Many existing works compare the accuracy of regressors trained on augmented radiomaps against baseline localization regressors (trained on non-augmented radiomaps) with default or weakly optimized hyperparameters. This makes it difficult to isolate the source of the reported improvement. Since data augmentation usually acts as a form of regularization [23], it may compensate for an under-optimized localization model rather than provide fundamentally new spatial information.

Moreover, modern generative models can be expensive to train and tune. VAEs, GANs, DDPMs (including tabular diffusion models) and DiTs require additional design choices, computational resources, and validation procedures. In practical indoor localization deployments, this additional cost is justified only if augmentation provides consistent and meaningful improvements over simpler alternatives, such as careful regressor selection and hyperparameter optimization.

These limitations motivate the present study. Rather than asking only whether synthetic fingerprints can improve localization accuracy, we investigate when such augmentation remains beneficial after strong baseline optimization. Specifically, we compare multiple generative models against carefully tuned localization regressors across controlled radiomap sparsity levels. This allows us to assess whether synthetic fingerprint augmentation provides robust gains or whether its benefits are limited to specific sparse-data regimes.

In summary, existing literature shows that generative augmentation can improve fingerprint-based localization in some settings. However, whether these gains persist against strong, optimized non-augmented baselines remains an open question. Addressing this question is the central objective of this paper.

## 3. Methodology

### 3.1. Farthest-Point Sampling for RP Omission

The radiomap topology and reference points (RPs) used to illustrate this subsection belong to the UTSIndoorLoc dataset [36,37], whose full details (fingerprint count, APs, preprocessing) are introduced in Section 4.1.1.

**Figure 1:**
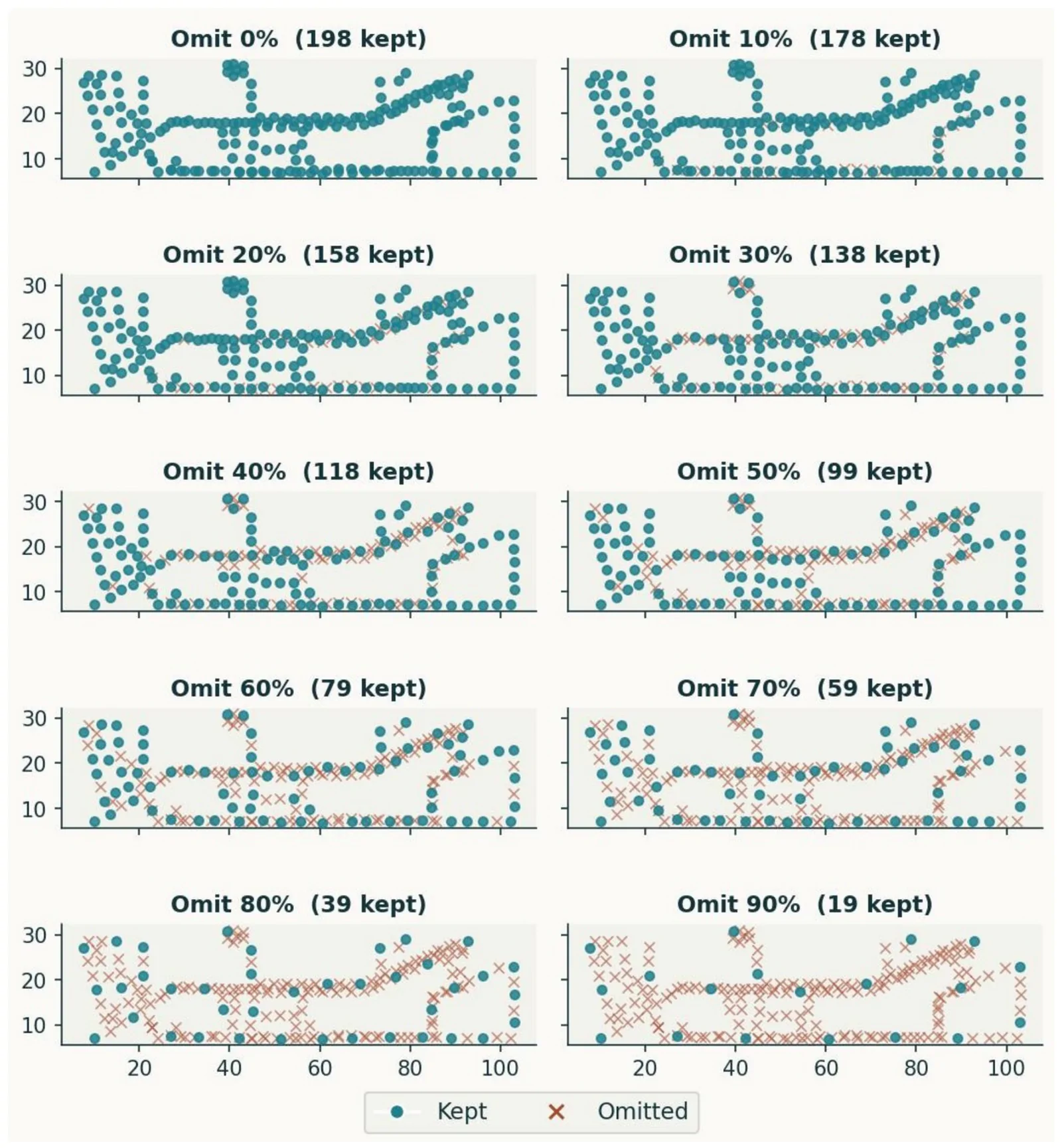
Radiomap for different omission rates [UTSIndoorLoc; dataset details in Section 4.1.1]. Teal dots represent kept RPs and red crosses represent omitted RPs.

To evaluate localization performance under sparse data conditions, some radiomap entries must be removed by omitting selected RPs and all fingerprints associated with them. Naïve random subsampling can create irregular and unrealistic coverage gaps, thereby confounding the effect of sparsity with spatial non-uniformity. To preserve approximately uniform spatial coverage across all omission levels, we adapt the farthest-point sampling algorithm [38] that iteratively removes the most spatially redundant RPs. The algorithm begins at the centroid of the 198 RPs and greedily chooses the point that is furthest away from all the selected RPs. It creates a ranking from the most spatially informative to the least. In order to create *p*% omission, the last ⌊p/100×198⌋ locations and their associated training samples are removed. Figure 1 visualizes the spatial distribution of kept and omitted RPs for each omission level.

### 3.2. Hyperparameter Optimization for Regression Models

To investigate whether careful model optimization can outweigh the benefits of complex data augmentation strategies, we systematically tune the hyperparameters of the baseline localization regressors—kNN, SVR, XGBoost, and DNN—using the Optuna framework [39,40], a widely used Bayesian optimization library that is substantially more sample-efficient than exhaustive grid search; the complete search space and tuning budget are reported in Appendix A. The performance evaluation of the trained regressors is discussed in Section 4. Table 1 presents the set of optimal hyperparameter settings for XGBoost, which was found to be the best-performing regressor on the UTSIndoorLoc dataset in most situations; see Section 4. Optimal settings for other regressors can be found in our GitHub repository [41].

### 3.3. Synthetic Fingerprint Augmentation

This subsection describes our end-to-end fingerprint augmentation pipeline. For each omission level, we (i) train a generative model on the available training fingerprints, (ii) sample a pool of synthetic RSSI vectors, (iii) apply multi-stage quality filtering to remove physically implausible and out-of-distribution (OOD) RSSI fingerprints, (iv) assign pseudo-coordinates to the remaining synthetic fingerprints, thereby converting them into synthetic radiomap entries (or generating them conditionally when the model supports it), and (v) train a localization regressor on the augmented radiomap and evaluate its performance. To mitigate generator overfitting, we periodically validate candidate checkpoints by measuring localization error on a held-out validation set and retain the best-performing checkpoint for final synthesis.

#### 3.3.1. Benchmarked Generative Models

To ensure a fair and reproducible comparison, the generative models were tuned through the complete augmentation pipeline, which includes synthetic fingerprint generation, quality filtering, pseudo-labeling, merging with real data, and downstream validation using the localization model. The reported configurations therefore correspond to the best-performing settings identified experimentally. Additional implementation details and configuration variants are available in our GitHub repository [41]. We benchmark the following five popular generative models:

- **VAE** [5]: Encoder–decoder MLP (256-128-64 latent) with the reparameterization trick, $\beta_{\mathrm{KL}}=0.001$, and 2000 epochs.
- **GAN** [7]: Generator (128-256-512-*D*) and discriminator (512-256-1) with Leaky ReLU and 2000 epochs.
- **DDPM** [10]: 4-layer MLP noise predictor with sinusoidal time embedding and self-conditioning [42], cosine noise schedule [43], T=200 timesteps, and 2000 epochs.
- **DiT** [35]: Diffusion transformer with patchification (13 patches of 16 dimensions), three transformer blocks with 8-head self-attention, EMA (γ=0.999), T=200 timesteps, and 2000 epochs.
- **TDPM** [12]: Conditional DDPM with a tabular U-Net architecture that conditions on spatial coordinates (x,y). It uses a cosine $\beta_{t}$ schedule, all-timestep loss aggregation, subtractive denoising, T=100 timesteps, and 200 epochs.

The first four models learn the mapping between RSSI values associated with different APs and generate synthetic RSSI fingerprints that are subsequently assigned pseudo-coordinates to form synthetic radiomap entries; pseudo-labeling will be explained in detail in Section 3.3.4. TDPM differs in that it directly models the relationship between RSSI values and spatial coordinates and therefore synthesizes fingerprints conditioned on the given coordinates, which enables an assessment of conditional models alongside the unconditional ones.

When trained for too long, generative models may overfit, thus producing less realistic fingerprints [10,22]. In order to mitigate this phenomenon, the training procedure is periodically stopped at predetermined points, i.e., every 100 epochs. At every break, 500 synthetic fingerprints are generated; they are filtered (Section 3.3.3), labeled with coordinates (Section 3.3.4), and combined with the real data. Then, the XGBoost regressor is once again trained, and its Mean Euclidean Error (MEE) is measured using a validation set. If the newly acquired MEE exceeds all previous values, the model state is saved as a checkpoint. After training completes, the checkpoint with the best MEE is restored and used for final fingerprint generation, which will be discussed in the following subsections.

#### 3.3.2. Synthesis Volume

The number of fingerprints generated is dictated by the omission rate in the radiomap. Let $N_{\mathrm{full}}$ be the number of real training fingerprints at 0% omission and $N_{\mathrm{real}}\left(q\right)$ the number of real training fingerprints after omitting q% of RPs. We set the target number of retained synthetic fingerprints as $N_{\mathrm{syn}}^{\mathrm{target}}\left(q\right)=\max\left(50,N_{\mathrm{full}}-N_{\mathrm{real}}\left(q\right)\right)$. The main idea is to bring the reduced training set close to the original size of the training set while keeping a minimal level of data augmentation (50 synthetic fingerprints) at low omission rates.

The models actually generate twice the target number of new raw fingerprints, as some of them are removed by the quality filters explained in Section 3.3.3. If more than $N_{\mathrm{syn}}^{\mathrm{target}}\left(q\right)$ synthetic fingerprints remain after filtering, only the first $N_{\mathrm{syn}}^{\mathrm{target}}\left(q\right)$ are retained to prevent the synthetic data from dominating the merged training set.

#### 3.3.3. Quality Filtering of Synthetic Fingerprints

Unfiltered outputs of generative models can contain physically unrealistic or out-of-distribution (OOD) RSSI vectors. If those unrealistic samples are included in the augmented training set, they introduce noise that may mislead the localization regression model, impacting its accuracy. To alleviate this, a multi-stage quality filtering pipeline is applied to retain only high-quality synthetic RSSI fingerprints:

1. **Clipping:** Generated RSSI values are clipped to the valid normalized range [0,1] to enforce physical admissibility.
2. **Sparsity mask:** To match the empirical sparsity of real fingerprints (only a limited number of APs are active at a location), each synthetic fingerprint keeps only its strongest AP readings, and the number it keeps is drawn from a clipped Gaussian fitted to the real active-AP counts. At omission level *q*, let $a_{i}^{\left(q\right)}$ denote the number of active APs (normalized RSSI above 0.01) in real training fingerprint *i*. From these counts we obtain the mean $\mu_{q}$, the standard deviation $\sigma_{q}$, the lower bound $\ell_{q}$ (the smallest observed count, floored at 3), and the upper bound $u_{q}$ (the 95th percentile, rounded down). For each synthetic fingerprint *j* we then draw
  (3) $$z_{j}∼N\;\left(\mu_{q},\sigma_{q}^{2}\right),\;m_{j}^{\left(q\right)}=\left\lfloor \mathrm{clip}\;\left(z_{j},\ell_{q},u_{q}\right)\right\rfloor ,$$
  retain the $m_{j}^{\left(q\right)}$ strongest AP values of that fingerprint, and set the remaining entries to zero. The four statistics $\mu_{q}$, $\sigma_{q}$, $\ell_{q}$ and $u_{q}$ are recomputed from the real training fingerprints still available at each omission level; this recomputation is the sense in which the mask adapts to sparsity. We write *m* for this activation count and reserve *K* for the neighbor count of the kNN gate below, as the two are unrelated.
3. **kNN gate:** A distribution-level check removes OOD samples. Distance here is the Euclidean distance between normalized RSSI vectors, not the distance between physical coordinates. Fixing K=5, we compute for every real training fingerprint the mean distance to its five nearest real RSSI neighbors, excluding the fingerprint itself, and set the threshold $\tau_{q}$ to the 90th percentile of those mean distances. A synthetic sample is retained only when its own five-neighbor mean distance falls below $\tau_{q}$. Only the numerical value of $\tau_{q}$ is recomputed at each omission level, so the gate follows the dispersion of the RSSI data actually available at that level rather than assuming one universal distance. No floor-plan, wall, or propagation information enters this step: the gate is an out-of-distribution test in RSSI space, not a test of geometric feasibility.

To evaluate whether these filters help or not, we conducted a small evaluation with two of the generative models, namely, GAN and VAE. Because training and re-sampling a generative model at every ratio is computationally expensive, we limited our evaluation to these two well-established generators. Appendix C reports a repeated-seed ablation of this quality-filtering pipeline. We conclude that the quality filter is beneficial in most cases.

#### 3.3.4. Pseudo-Labeling the Synthetic Fingerprints

Pseudo-coordinates for filtered synthetic fingerprints are predicted using the previously trained baseline regression model, thereby forming synthetic radiomap entries [8,9,11]. We use XGBoost for this step because it is the strongest of the four tuned regressors, attaining the lowest error at most of the sparsity levels (Table 2). The process is as follows:

- Train the baseline XGBoost regressor on the non-omitted fingerprints from the current radiomap. Its hyperparameters are fine-tuned as explained in Section 3.2.
- Filter fingerprints based on their quality according to Section 3.3.3 and then feed each remaining synthetic fingerprint to the trained regressor to obtain its pseudo-coordinate: $\hat{p}=\left(\hat{x},\hat{y}\right)$.
- Add the resulting pseudo-labeled fingerprints to the radiomap as synthetic radiomap entries. The augmented radiomap now contains both real and synthetic entries.
- Train a new XGBoost regressor on the augmented radiomap and evaluate its accuracy on the held-out test set. This regressor is then used as part of our evaluation (see Section 4.1.3) to assess the effect of synthetic fingerprints on localization accuracy.

**Table 2:** Localization mean Euclidean error (in meters) produced by four regressors for different omission levels. The best value at each omission level is shown in bold.

Regressor	0%	10%	20%	30%	40%	50%	60%	70%	80%	90%
kNN	2.50	2.69	2.92	4.42	4.89	4.91	**4.65**	**5.93**	**7.02**	**9.03**
SVR	6.62	6.77	6.97	7.13	7.07	7.11	7.55	8.60	8.55	11.59
XGBoost	**1.66**	**2.33**	**2.78**	**3.53**	**3.83**	**4.30**	5.57	7.13	7.23	9.96
DNN	3.84	4.33	4.42	4.91	7.81	5.74	6.66	10.58	11.51	15.04

Because no oracle location is available for synthetic fingerprints, pseudo-labeling necessarily inherits the error of the labeling regressor, which can itself exceed 5 m under high sparsity (Table 2). We treat this as an inherent limitation of pseudo-labeling rather than a fixable implementation issue; Section 4.1.5 reports a dedicated check of its impact on our conclusions, and we note that TDPM sidesteps the issue entirely by conditioning directly on coordinates instead of requiring pseudo-labels.

## 4. Performance Evaluation

### 4.1. Performance Evaluation for the UTSIndoorLoc Dataset

#### 4.1.1. Dataset, Preprocessing, and Feature Selection

We use the UTSIndoorLoc, a publicly available dataset of 522 labeled fingerprints collected at 198 RPs in a single-floor indoor setting [36,37]. The measurements include 206 APs and 2D spatial coordinates of the RPs. While most RPs contain a single fingerprint, some have more than one.

For missing APs in fingerprints, we used an RSSI value of −105 dBm, which is substantially lower than the normal receiver sensitivity. The RSSI values are then normalized to the fixed range [0,1] to place all of them on a common scale and improve the stability and convergence of the learning algorithms:

(4) $$r_{\mathrm{norm}}=\frac{r_{\mathrm{dBm}}+105}{75}$$

where −105 dBm and −30 dBm correspond to 0 and 1, respectively. The training (60%), validation (20%), and test (20%) sets are generated using stratified random sampling, where fingerprints are grouped by reference point (RP) to ensure that repeated measurements from the same RP are assigned consistently and that the spatial coverage of the radiomap is preserved across all sets.

To reduce the dimensionality of the data, i.e., the number of APs (features), we applied a 5% occurrence filter that removes APs detected in fewer than 5% of training samples, which resulted in retaining 125 APs (features).

#### 4.1.2. Performance Evaluations of Regression Models

Table 2 reports the localization MEE for four different regressors at different omission levels; the regressors were trained without any synthetic data. At 0% omission, XGBoost achieved the best performance with an MEE of 1.66 m, substantially outperforming kNN (2.50 m), DNN (3.84 m), and SVR (6.62 m). XGBoost remained the best model from 0% through 50% omission, with error increasing from 1.66 m to 4.30 m as the radiomap became progressively thinner.

Starting from a 60% omission level, kNN’s performance becomes better than that of any other regressor. For example, kNN is the best at 60% omission with 4.65 m versus 5.57 m with XGBoost, and this is maintained at 70%, 80%, and 90% omission levels. We can therefore conclude that the dominating regressor is dependent on the density regime: XGBoost will work better when there is enough spatial support, and kNN will be more effective when the training fingerprints are highly sparse.

SVR is the weakest at most rates of omission, while DNN is the most unstable. Errors are strongly right-skewed at every omission level (median ≪ mean, long P90 tail), e.g., XGBoost at 0%: median = 0.07 m vs. mean = 1.66 m. The per-sample error distributions and the full percentile statistics are deferred to Appendix B.

#### 4.1.3. Performance Evaluation of Data Augmentation

**Figure 2:**
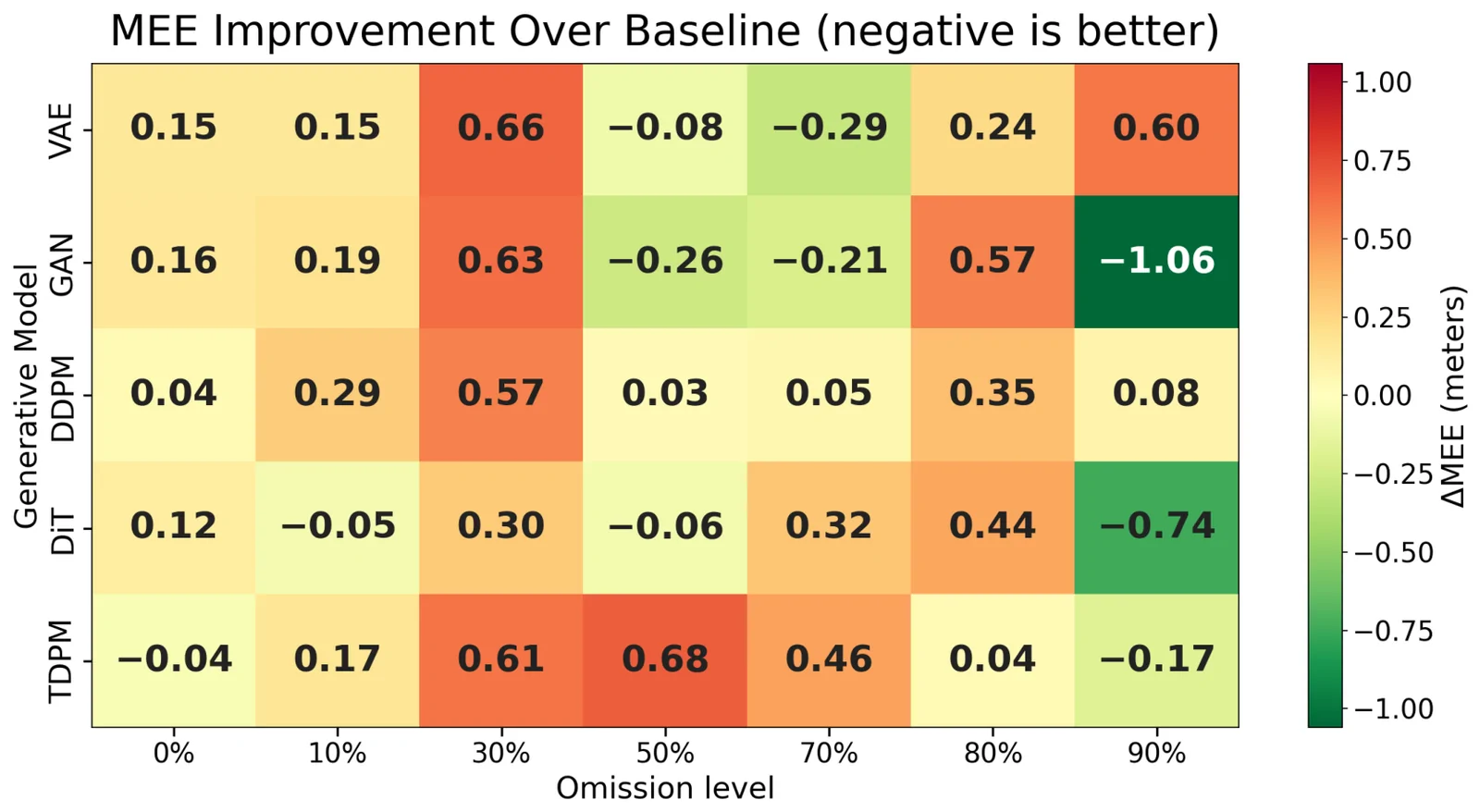
Improvement over baseline regressor (XGBoost from Table 2) in terms of difference in MEE, i.e., ${\mathrm{MEE}}_{\mathrm{aug}.\;\mathrm{model}}-{\mathrm{MEE}}_{\mathrm{XGBoost}}$. Negative (green) values indicate that data augmentation improves localization accuracy relative to the non-augmented XGBoost regressor; each cell is the corresponding difference between two entries of Table 3. Only 10 of the 35 cells are negative, and the improvement exceeds 0.5 m in just two of them.

In this subsection, we determine how the synthetic radiomap augmentation affects localization accuracy. Table 3 presents the localization error for five generative models. For convenience, we also include Figure 2, which provides a compact summary of the improvements and degradations brought by generative models: green cells indicate improvement over the non-augmented baseline XGBoost from Table 2 (negative ΔMEE), while red cells indicate degradation. Because training generative models on cloud computing infrastructure is expensive, we were only able to train them at a subset of sparsity levels. This choice allowed us to span a range of sparsity settings while keeping the computational cost at a manageable level.

At smaller omission levels, i.e., when the radiomap is still dense, augmentation does not add much value and can even be detrimental. For example, at 0% omission, the best result is produced by TDPM, while the second best is the non-augmented baseline regressor, with only 0.04 m more average error.

The situation is very similar at omission levels of 10%, 30%, and 50%. At 10% DiT produced the best average accuracy, with the baseline regressor the runner-up by only 0.05 m, while at 30% no generator matches the non-augmented XGBoost baseline at all: the best of them, DiT, is 0.30 m worse. GAN achieves the best result at 50% omission, which is better than the baseline by only 0.26 m.

Measured against the XGBoost baseline alone, augmentation appears to help in the severe-sparsity regimes: at 70% the VAE is better by 0.29 m and at 90% GAN gains 1.06 m, although at 80% even this comparison fails—the best generator, TDPM, is 0.04 m worse than the non-augmented XGBoost.

This impression does not survive a comparison against the best non-augmented regressor, however. As established in Section 4.1.2, XGBoost ceases to be the strongest baseline beyond 60% omission; kNN is. Comparing each augmented pipeline against whichever baseline is stronger at that sparsity level, the non-augmented kNN outperforms every augmented model at both 70% (5.93 m, versus 6.84 m for the best generator) and 80% (7.02 m versus 7.27 m), and only at 90% does an augmented model overtake it, by 0.13 m (GAN, 8.90 m versus 9.03 m). Both baselines are shown directly in Table 3. The apparent severe-sparsity benefit of augmentation is largely an artifact of measuring against a regressor that has itself become suboptimal in that regime—precisely the methodological pitfall this paper set out to examine.

Within the dense regime, entries are mostly near zero or positive, i.e., augmentation contributes marginal improvements or can even be detrimental. The largest negative values in Figure 2 occur between 70% and 90% omission, particularly for VAE, GAN, DiT, and TDPM. That heatmap is, however, computed against the XGBoost baseline; once kNN is admitted, the apparent gains at 70–80% disappear entirely. This supports our primary hypothesis in an even stronger form than anticipated: synthetic augmentation is not a universal tool that unconditionally improves localization performance, and once each sparsity regime is compared against its own best non-augmented regressor, no omission level yields a gain larger than 0.26 m (at 50% omission).

We can conclude from the above analysis that no augmentation model consistently beats the baseline across different omission levels. Most of the time, the non-augmented and properly tuned baseline regressor either wins over data-augmented models or is only slightly behind the best augmented result, with a very marginal difference.

#### 4.1.4. Assessment of the Synthesis Volume

To assess how the proportion of synthetic data affects localization accuracy, we generated synthetic fingerprints at 25%, 50%, 100%, 150%, and 200% of the target volume $N_{\mathrm{syn}}^{\mathrm{target}}\left(q\right)$ (Section 3.3.2) for VAE and GAN across several omission levels. Because training and re-sampling a generative model at every ratio is computationally expensive, we limited our evaluation to these two well-established generators). Results are shown in Table 4. No single ratio wins consistently across omission levels or generators, and more synthetic data is not monotonically better. Averaged across all (omission level, generator) cells, the lowest mean MEE occurs at 25% synthetic volume (6.56 m), compared with 6.72–6.99 m at 50–200%; but 25% is not a universal winner, being optimal in only 3 of the 10 cells, with 100% optimal in a further 3 and larger ratios preferred in the remaining 4. We therefore recommend a conservative 25–100% of $N_{\mathrm{syn}}^{\mathrm{target}}\left(q\right)$ rather than assuming that larger synthetic pools are better. This finding also replicates on a second, structurally different dataset (Section 4.2).

#### 4.1.5. Circularity Check for the Pseudo-Labeling Regressor

**Figure 3:**
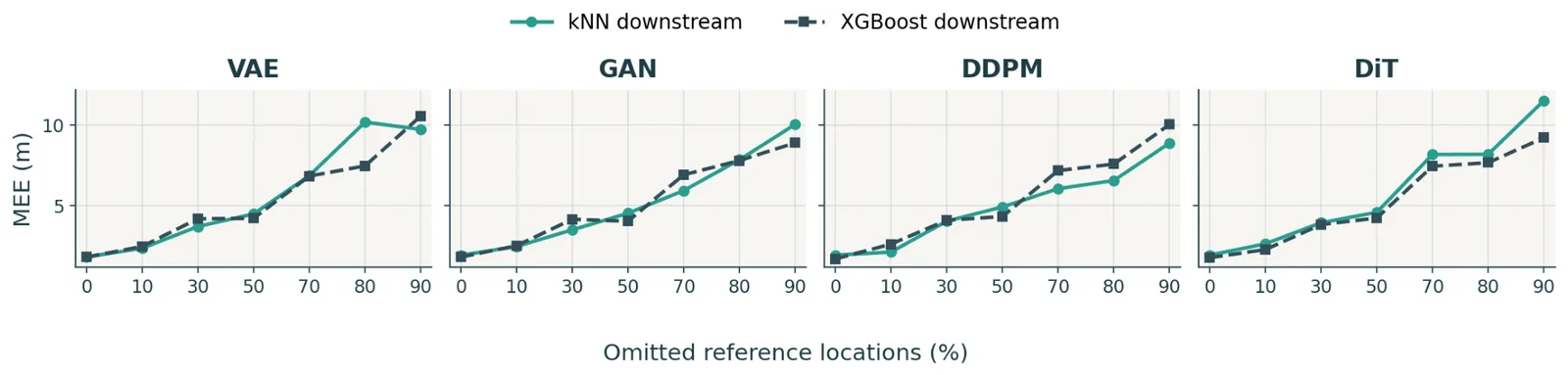
UTSIndoorLoc localization MEE versus omitted RPs, comparing kNN and XGBoost as the downstream (evaluation) regressor for each generator, with the XGBoost pseudo-labeler held fixed.

Because XGBoost is used both to generate pseudo-coordinates for synthetic fingerprints (Section 3.3.4) and to evaluate localization accuracy in this section, the “augmentation ≈ neutral” finding could in principle be an artifact of reusing the same regressor for both roles. To rule this out, we repeated the augmentation evaluation using kNN as the downstream localizer instead of XGBoost, while keeping the XGBoost pseudo-labeler unchanged (Figure 3, shown for the four pseudo-labeled generators). The ranking and conclusions are essentially unchanged. The two downstream regressors agree closely at low-to-moderate sparsity—within 0.5 m in 14 of the 16 cells up to 50% omission—and diverge more at high sparsity, by as much as 2.7 m at 80%. Crucially, however, the disagreement has no systematic direction: XGBoost is the better downstream regressor in 17 of the 28 cells and the worse in 11. A genuine circularity artifact would favor the reused regressor consistently, whereas what we observe is scatter, confirming that the neutral-to-marginal effect of augmentation is not an artifact of regressor reuse.

Two details of Figure 3 deserve mention. First, TDPM is excluded from the comparison because it is coordinate-conditional and uses no pseudo-labeling regressor (Section 3.3.4); there is consequently no labeler–evaluator coupling for it to test, and it is instead examined separately in Table 5. Second, the DDPM curve for the kNN downstream regressor coincides exactly with the non-augmented kNN baseline at 0, 10, 50, 70 and 90% omission, because at those levels the quality gate of Section 3.3.3 retained no synthetic samples and the training set was therefore left unaugmented.

#### 4.1.6. Comparison of Conditional and Pseudo-Labeled TDPM

As a further check, we compare the pseudo-labeled TDPM (using XGBoost-generated pseudo-coordinates) against a genuinely conditional TDPM variant that requires no pseudo-labels at all (Table 5). Two comparisons must be kept apart here. Against the pseudo-labeled variant, conditioning helps at four of the seven omission levels (0%, 30%, 70% and 80%), so dispensing with pseudo-labels is mildly preferable to relying on them. Against the non-augmented baseline, however, the conditional variant wins at only two levels (0% and 90%), missing by just 0.04 m at 80%. Conditioning therefore removes the dependence on an external labeling regressor—addressing the concern raised above—but does not by itself yield a consistent accuracy advantage over performing no augmentation at all. This reinforces that no augmentation approach, conditional or pseudo-labeled, offers a reliable and universal benefit.

### 4.2. Generalization to UJIIndoorLoc Dataset

**Figure 4:**
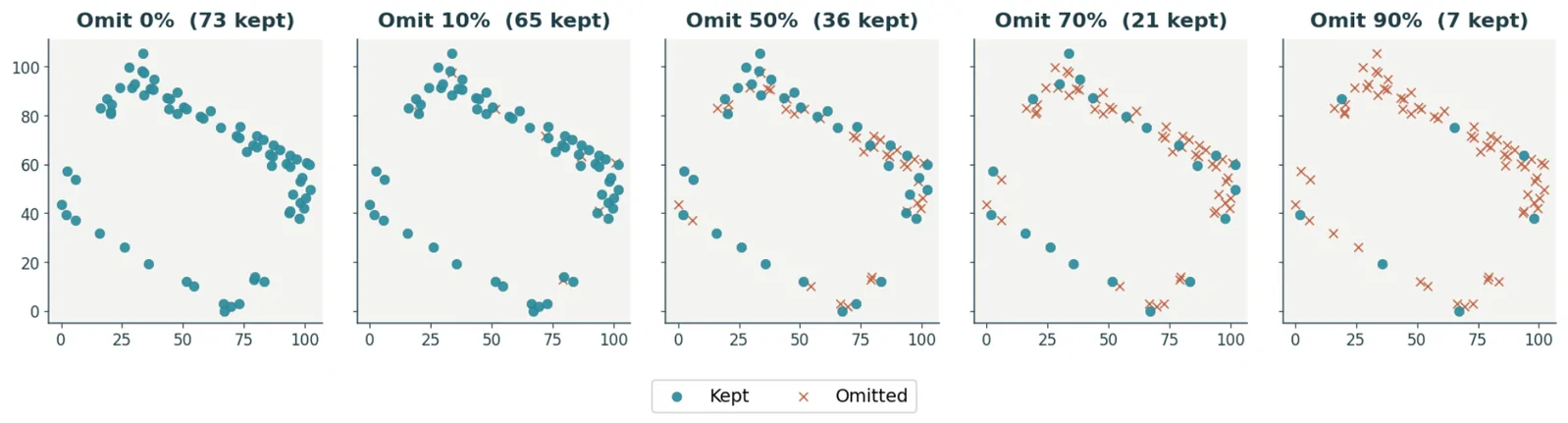
Farthest-point sparsification of the second dataset (UJIIndoorLoc building 1, floor 2; 1577 fingerprints over 73 RPs spanning approximately 102×106 m), i.e., the counterpart of Figure 1 for UTSIndoorLoc. Teal dots are RPs kept for training and red crosses those omitted; panels show five of the seven omission levels, with 73, 65, 36, 21 and 7 of the 73 locations retained at 0%, 10%, 50%, 70% and 90% omission respectively. Axes are in meters and share the same extent across panels.

To assess whether our findings generalize beyond UTSIndoorLoc, we repeated the synthesis-volume sweep and the augmentation-vs-baseline comparison on UJIIndoorLoc [44] building 1, floor 2 (Figure 4)—a single-floor radiomap with 1577 fingerprints, 149 APs, and 73 RPs spanning approximately 102×106 m, which has a substantially denser per-location sampling rate and a different spatial layout than UTSIndoorLoc.

We begin with the non-augmented regressors. Table 6 reports all four of them on this dataset, the counterpart of Table 2. The density-dependent behavior observed on UTSIndoorLoc is even more pronounced here: kNN is the strongest regressor at every omission level, and XGBoost—which was the best regressor on UTSIndoorLoc up to 50% omission—trails it by between 1.7 and 9.3 m throughout. The finding of Section 4.1.2 that the dominant regressor depends on the density regime therefore also generalizes, and it is the reason kNN serves as both the baseline and the downstream regressor in the augmentation experiments that follow.

**Figure 5:**
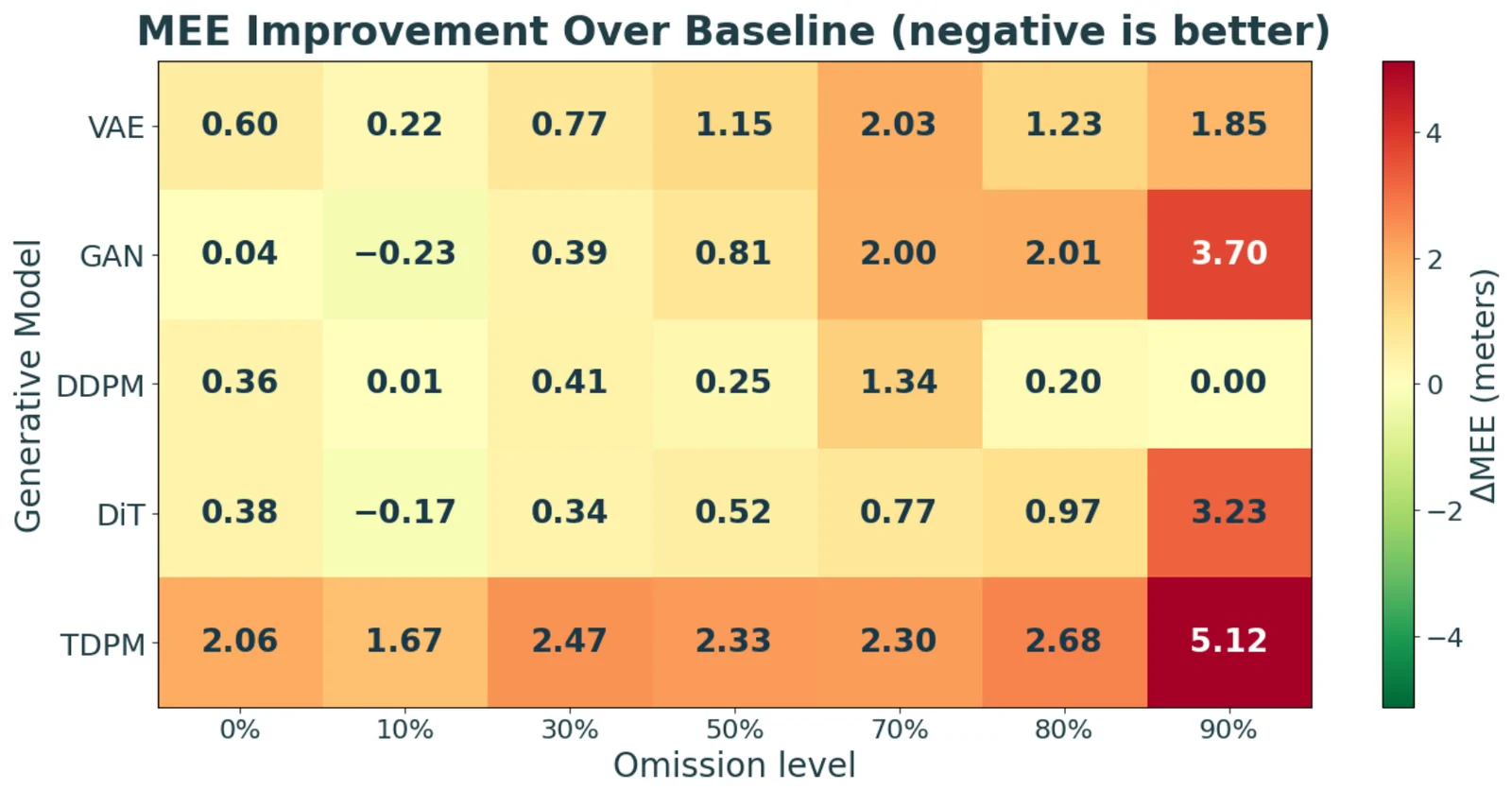
UJIIndoorLoc building 1: improvement over the non-augmented kNN baseline of Table 6 (analogous to Figure 2). Each cell is the corresponding difference between two entries of Table 7. Almost every cell is positive (red), i.e., augmentation degrades accuracy; the only negative (improving) cells are GAN (−0.23 m) and DiT (−0.17 m) at 10% omission.

The augmentation pipeline is otherwise unchanged: pseudo-coordinates are assigned by XGBoost exactly as in Section 3.3.4, while both the baseline and the downstream localizer are kNN. Because the pseudo-labeling and evaluation regressors therefore differ, these results are by construction free of the circularity examined in Section 4.1.5. The same non-augmented kNN result serves as the baseline throughout—in Table 6 and Table 7, and in Figure 5—so that every comparison on this dataset is made against a single reference.

Turning to augmentation, both central findings replicate on this second dataset. First, no single synthetic fingerprint volume dominates across omission levels or generators (Table 8): the smaller ratios of 25% and 50% are optimal in 8 of the 10 (omission level, generator) cells, but the remaining two are best at 200%, so the conservative 25–100% recommendation of Section 4.1.3 carries over rather than sharpening into a single preferred value. Second, augmentation beats the non-augmented kNN baseline in only 2 of the 35 (generator, omission-level) cells (Table 7 and Figure 5), both at 10% omission and both by less than 0.25 m. The picture on this second dataset is therefore even less favorable to augmentation than on UTSIndoorLoc.

### 4.3. Discussion

The findings present a more nuanced picture than is sometimes suggested in the literature, where generative fingerprint augmentation is believed to provide substantial benefits. As we witnessed in our experiments, no augmentation model consistently beats the non-augmented baseline regressor across different omission levels. Most of the time, the non-augmented and properly tuned baseline regressor is only slightly behind the best data-augmented model, with only a modest difference. On the second dataset the picture is even less favorable to augmentation: no generator improves on the tuned baseline at any omission level except two marginal cases at 10% omission.

In addition, training generative models can be very expensive since they require a lot of computational resources, time, and specialized hardware (e.g., expensive graphical processing units). In contrast, regression localization models can be trained locally on a cheap laptop within a very short time.

Our findings suggest a clear practical guideline: practitioners should first invest in regression model selection for localization and hyperparameter optimization. Generative fingerprint augmentation should be considered only after regressor selection and hyperparameter optimization have been exhausted, and even then only where a sub-meter gain would justify the cost of training and validating several generative models. Notably, none of the sparsity levels we examined met that condition once each was compared against its own best non-augmented regressor. A further practical consequence of Section 4.1.2 is that the choice of regressor should itself be made per density regime: XGBoost dominates when spatial support is adequate, whereas kNN becomes preferable once the radiomap is severely thinned.

A key limitation of this analysis is that both datasets are single-floor (UTSIndoorLoc and UJIIndoorLoc building 1). Whether these findings extend to true multi-floor or multi-building environments with substantially higher AP density and larger spatial extent remains an open question for future work. We also did not re-ablate the 5% AP-occurrence filter, which is dataset-specific and may require re-tuning where AP density differs substantially.

## 5. Conclusions

We revisited Wi-Fi RSSI fingerprinting with the goal of clarifying when generative radiomap augmentation is actually justified. Building on the existing work on fingerprint-based localization and common regressors, we introduced a controlled sparsification protocol based on farthest-point thinning and evaluated four localization model families (kNN, SVR, DNN, and XGBoost) together with five generative models (VAE, GAN, DDPM, DiT, and TDPM) to augment data across multiple omission levels.

The performance evaluation shows that careful baseline optimization is a dominant factor: in the dense and moderately sparse regimes (0–50% omission) the tuned XGBoost localizer stays within 0.26 m of the best augmented pipeline at every level, and at 30% omission it beats every one of them, so augmentation buys at most a fraction of a meter over a well-optimized regressor. Crucially, the apparent benefit of augmentation under extreme sparsity (≥70% omission) does not survive comparison against the strongest non-augmented regressor for that regime: at 70% and 80% omission the tuned kNN baseline outperforms every augmented pipeline, and at 90% the best generator leads it by only 0.13 m. Measured against the best baseline available at each sparsity level, the largest gain from augmentation anywhere in our experiments is 0.26 m, obtained at 50% omission. On a second, structurally different building (UJIIndoorLoc building 1), augmentation improved on the tuned kNN baseline in only 2 of the 35 (generator, omission-level) cells, indicating that even these conditional gains do not transfer reliably across environments.

Overall, the results support a conditional view of augmentation: synthetic radiomap augmentation should be considered a last resort after baseline model optimization, and its value must be validated for each model and sparsity level rather than assumed.

In the future, our hypothesis should be validated in more generalized settings, e.g., larger and multi-floor datasets across multiple buildings. Further work should also explore more principled conditional generation methods for all model classes, including coordinate-conditional diffusion and adversarial models, in order to reduce reliance on pseudo-labeling. In addition, modeling temporal RSSI drift and evaluating augmentation under domain shift remain important directions for future research. Finally, data-driven decision rules could be developed to predict when augmentation is likely to be beneficial and to determine the most suitable generator and synthesis level accordingly.

## Figures and Tables

**Table 1:** Optimal hyperparameters for the baseline XGBoost regressor.

Parameter	Value
n_estimators	250
max_depth	7
learning_rate	0.0924
subsample	1.0
colsample_bytree	1.0
reg_alpha	0
reg_lambda	1
tree_method	hist

**Table 3:** Localization mean Euclidean error (in meters) of the XGBoost regressor trained on augmented radiomaps produced by five generative models, together with both non-augmented baselines carried over from Table 2. The best value at each omission level—including the baselines—is shown in bold. Note that XGBoost is not the strongest non-augmented regressor beyond 60% omission: the tuned kNN baseline outperforms every augmented pipeline at both 70% and 80% omission.

Method\Omission	0%	10%	30%	50%	70%	80%	90%
Baseline kNN	2.50	2.69	4.42	4.91	**5.93**	**7.02**	9.03
Baseline XGBoost	1.66	2.33	**3.53**	4.30	7.13	7.23	9.96
VAE	1.81	2.48	4.19	4.22	6.84	7.47	10.56
GAN	1.82	2.52	4.16	**4.04**	6.92	7.80	**8.90**
DDPM	1.70	2.62	4.10	4.33	7.18	7.58	10.04
DiT	1.78	**2.28**	3.83	4.24	7.45	7.67	9.22
TDPM	**1.62**	2.50	4.14	4.98	7.59	7.27	9.79

**Table 4:** Sensitivity of localization MEE (m) to synthesis volume, expressed as a percentage of the target number of synthetic fingerprints $N_{\mathrm{syn}}^{\mathrm{target}}\left(q\right)$ (Section 3.3.2), for VAE and GAN across omission levels. The Baseline column repeats the non-augmented XGBoost error at that omission level (Table 2) for reference; bold marks the best ratio for each generator.

Omit %	Gen	Baseline	25%	50%	100%	150%	200%
30	VAE	3.53	4.484	4.520	4.404	**4.362**	4.434
30	GAN	3.53	**3.901**	4.199	4.509	4.828	4.776
50	VAE	4.30	4.285	4.439	4.345	4.343	**4.173**
50	GAN	4.30	4.292	4.699	4.119	4.110	**4.023**
70	VAE	7.13	**6.793**	7.008	7.437	8.065	7.822
70	GAN	7.13	7.256	7.370	7.210	**7.032**	7.072
80	VAE	7.23	7.799	7.710	**7.463**	7.862	8.024
80	GAN	7.23	8.018	7.918	**7.566**	7.913	7.904
90	VAE	9.96	9.456	9.809	**9.286**	10.638	10.948
90	GAN	9.96	**9.353**	9.595	10.812	10.194	10.711

Note: this sweep is an independent re-run of the augmentation pipeline, in which the generative models were retrained. Because generator training is stochastic, the 100% column—although nominally the same synthesis volume $N_{\mathrm{syn}}^{\mathrm{target}}\left(q\right)$ used in Table 3—does not reproduce the corresponding entries of that table exactly, differing by between 0.01 m and 1.9 m. The five ratios within each row are drawn from a single consistent run, so the across-ratio comparisons that this table is intended to support remain valid; individual cells should not, however, be compared across Table 3 and this table.

**Table 5:** Truly conditional TDPM (no XGBoost pseudo-labels) versus the pseudo-labeled TDPM variant, with the non-augmented XGBoost baseline of Table 2 repeated for reference. MEE in meters. Two separate comparisons are marked: **bold** indicates the better of the two TDPM variants at that omission level, while ^†^ marks the cells in which the conditional variant also improves on the baseline. The conditional variant wins the first comparison at four omission levels but the second at only two.

Omit %	Baseline (XGBoost)	TDPM-Cond	TDPM-Pseudo
0	1.66	**1.62** ^†^	1.85
10	2.33	2.50	**2.24**
30	3.53	**4.14**	4.31
50	4.30	4.98	**4.12**
70	7.13	**7.59**	7.96
80	7.23	**7.27**	7.58
90	9.96	9.79 ^†^	**9.76**

**Table 6:** UJIIndoorLoc building 1: localization mean Euclidean error (in meters) of the four non-augmented regressors, the counterpart of Table 2 for the second dataset (N=316 test fingerprints). The best value at each omission level is shown in bold; unlike on UTSIndoorLoc, kNN is the strongest regressor at every omission level.

Regressor	0%	10%	30%	50%	70%	80%	90%
kNN	**1.38**	**2.06**	**2.21**	**3.66**	**5.43**	**6.96**	**11.46**
SVR	8.52	8.98	9.51	9.81	10.54	12.62	19.06
XGBoost	3.05	3.92	3.97	5.65	7.65	11.69	20.79
DNN	4.31	5.29	5.58	6.64	9.68	17.80	23.85

**Table 7:** UJIIndoorLoc building 1: localization MEE (m) of the kNN-downstream regressor trained on radiomaps augmented by five generative models, analogous to Table 3 for UTSIndoorLoc. **Bold** indicates the generator beats the non-augmented baseline at that omission level.

Omit %	Baseline	VAE	GAN	DDPM	DiT	TDPM
0	1.377	1.978	1.415	1.738	1.757	3.438
10	2.055	2.271	**1.824**	2.062	**1.881**	3.727
30	2.211	2.984	2.605	2.622	2.547	4.682
50	3.664	4.812	4.477	3.912	4.189	5.998
70	5.427	7.459	7.425	6.763	6.196	7.728
80	6.957	8.187	8.964	7.156	7.927	9.642
90	11.461	13.307	15.161	11.461 *	14.687	16.584

* At 90% omission the quality gate retained no DDPM samples, so this entry is the unaugmented result rather than an augmented one, which is why it coincides exactly with the baseline.

**Table 8:** UJIIndoorLoc building 1: sensitivity of localization MEE (m) to synthesis volume, the counterpart of Table 4 for the second dataset. Bold marks the best ratio for each generator.

Omit %	Gen	25%	50%	100%	150%	200%
30	VAE	**3.868**	3.951	4.658	5.024	5.090
30	GAN	**3.703**	4.090	4.798	4.567	4.646
50	VAE	**5.355**	5.438	5.561	5.542	5.636
50	GAN	5.878	**5.566**	5.700	5.610	5.631
70	VAE	**7.719**	8.032	7.801	7.808	7.965
70	GAN	8.019	8.338	8.195	7.992	**7.805**
80	VAE	12.444	12.358	12.699	12.250	**11.899**
80	GAN	13.050	**12.860**	13.400	13.180	13.310
90	VAE	20.844	**19.962**	20.817	20.872	22.478
90	GAN	21.950	**21.700**	22.300	22.150	22.550

**Figure A1:**
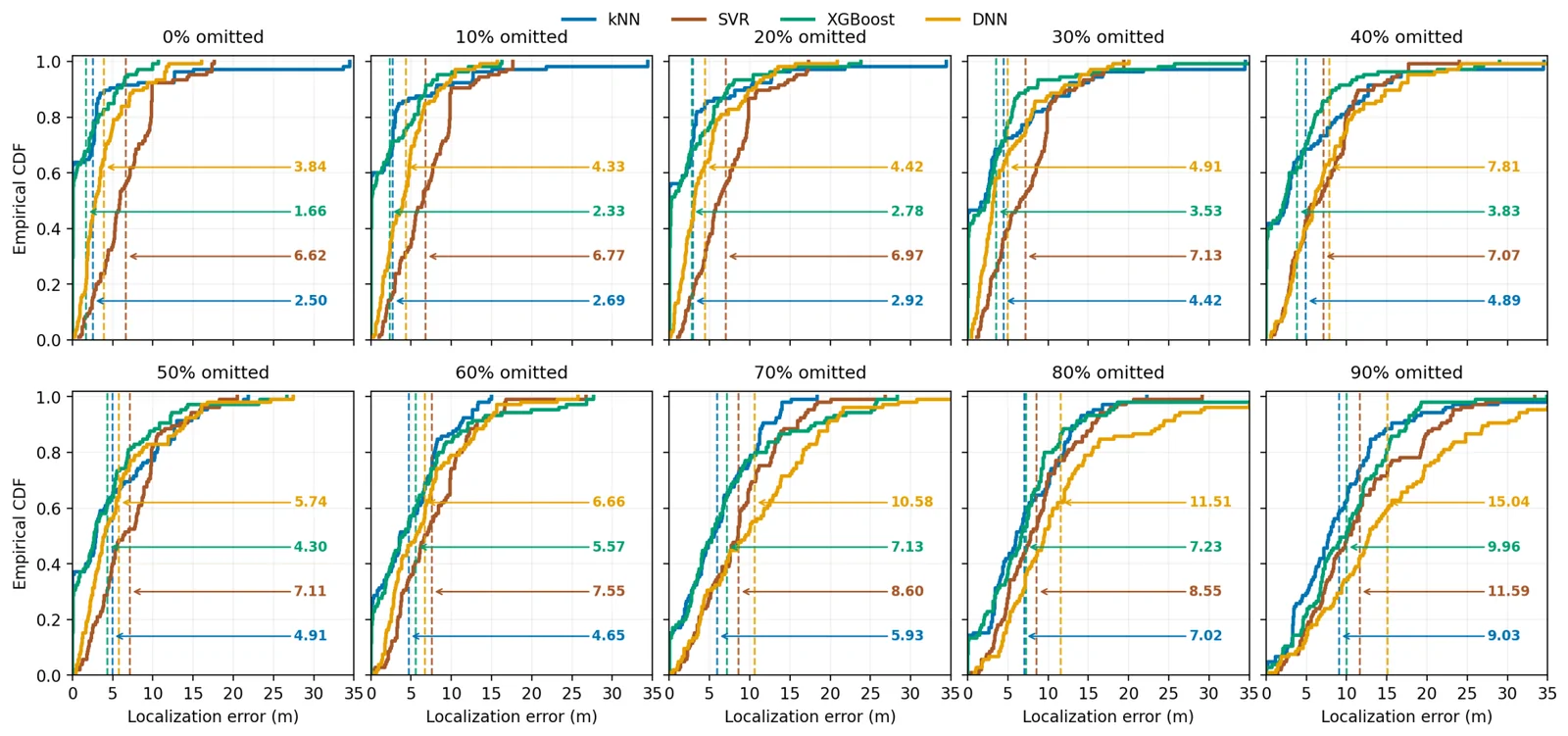
CDF of localization error by regressor and omission level for the non-augmented baselines of Table 2; dashed vertical lines and the annotated values mark the mean of each regressor.

## Data Availability

The UTSIndoorLoc dataset used in this study is publicly available at IEEE DataPort [37], and the UJIIndoorLoc dataset is publicly available from the UCI Machine Learning Repository [44]. Code and additional hyperparameter configurations are available at [41].

## Appendix A. Hyperparameter Tuning Details

**Table A1:** Complete hyperparameter search space used for Optuna tuning. Tuning uses Optuna’s TPE (Tree-structured Parzen Estimator) sampler with a fixed seed (42), minimizing the mean validation localization error (MEE). An independent study of 10 trials is run for each regressor × feature track (Full/tree-selected) × omission level—i.e., the per-model search budget is 10 iterations.

Model	Hyperparameter	Type	Search Range/Choices
kNN	n_neighbors	int	1–15
kNN	weights	categorical	{uniform, distance}
SVR (RBF, per-axis)	C	categorical	{0.1, 1, 10, 100}
SVR (RBF, per-axis)	gamma	categorical	{scale, 0.01, 0.1}
XGBoost	n_estimators	int (step 50)	100–300
XGBoost	max_depth	categorical	{3, 5, 7}
XGBoost	learning_rate	float (log)	0.01–0.1
DNN	lr	float (log)	$10^{-4}$–$10^{-3}$
DNN	dropout	float	0.1–0.3

## Appendix B. Error-Distribution Statistics for the Baseline Regressors

Table A2 reports the full per-sample error distribution underlying Table 2 and Figure A1, computed over N=105 test fingerprints. Percentiles follow the sort-and-index rule (e.g., p90 is the error at index ⌈0.9N⌉ of the sorted per-sample errors). The statistics confirm that localization errors are strongly right-skewed rather than symmetric: at low omission the median is near zero for kNN and XGBoost while the mean is 1.6–2.5 m, and the p90 tail extends to 3–5× the median. Consequently, the mean values in Table 2 are driven by a minority of large-error test points, and standard deviations are comparable to or larger than the means at low sparsity.

The near-zero mass of kNN and XGBoost in Figure A1 has two distinct causes. The tuned kNN is a 1-NN regressor, so it copies the coordinate label of a single stored fingerprint and returns exactly zero whenever that neighbor lies at the test point’s own RP. XGBoost instead predicts continuous coordinates and produces no error below $10^{-9}$ m, but 58 of its 105 errors are at most 0.10 m and are therefore indistinguishable from zero at this scale. Exact retrieval is frequent because the partition is drawn over individual fingerprints rather than over RPs, and UTSIndoorLoc holds 2.64 measurements per RP on average, so 76 of the 105 test fingerprints leave a sibling measurement of their own RP in the training set. This also reconciles the zero mass with the 34.445 m maximum: those 76 points average 0.48 m, whereas the 29 whose RP was not retained average 7.78 m, since a 1-NN predictor can then only answer with some other RP’s coordinates. The kNN curve is thus a mixture of near-exact retrieval and outright failure whose mean of 2.50 m describes neither regime. This is a property of the evaluation protocol rather than of any augmentation method: every regressor and pipeline in this paper uses the identical partition, so all comparisons between them are unaffected.

**Table A2:** Localization error distribution by regressor and omission level (N=105 test fingerprints): mean, standard deviation, and the 10th, 50th (median), and 90th percentiles, all in meters.

Regressor	Omit %	Mean	Std	p10	p50	p90
kNN	0	2.497	6.217	0.000	0.000	4.688
kNN	10	2.685	5.839	0.000	0.000	8.143
kNN	20	2.920	5.876	0.000	0.000	9.336
kNN	30	4.416	7.046	0.000	2.117	12.701
kNN	40	4.892	7.112	0.000	2.639	12.701
kNN	50	4.914	5.634	0.000	2.937	12.951
kNN	60	4.650	4.207	0.000	3.583	11.149
kNN	70	5.930	4.476	0.000	5.539	11.624
kNN	80	7.019	5.102	0.000	6.050	13.621
kNN	90	9.029	6.828	2.667	7.783	15.661
SVR	0	6.625	3.826	2.158	5.890	9.879
SVR	10	6.771	3.931	1.952	6.510	10.006
SVR	20	6.972	4.051	2.073	6.176	12.507
SVR	30	7.132	4.360	1.943	6.594	13.329
SVR	40	7.072	4.535	2.096	6.629	13.370
SVR	50	7.111	4.375	2.020	6.323	13.379
SVR	60	7.551	4.550	2.525	6.721	13.194
SVR	70	8.596	4.966	2.498	8.583	15.946
SVR	80	8.547	4.998	3.214	7.781	15.441
SVR	90	11.592	6.845	4.151	10.114	21.827
XGBoost	0	1.656	2.582	0.008	0.072	5.491
XGBoost	10	2.328	3.531	0.010	0.112	6.811
XGBoost	20	2.781	4.354	0.025	0.260	7.175
XGBoost	30	3.527	5.643	0.016	2.559	7.504
XGBoost	40	3.834	5.484	0.017	2.311	8.647
XGBoost	50	4.298	5.173	0.016	2.693	10.815
XGBoost	60	5.575	6.175	0.048	3.931	12.041
XGBoost	70	7.133	6.889	0.053	5.366	18.097
XGBoost	80	7.232	6.488	0.038	6.729	13.541
XGBoost	90	9.961	5.865	2.312	9.969	17.412
DNN	0	3.842	3.121	0.908	2.851	8.850
DNN	10	4.330	3.087	0.975	3.979	8.627
DNN	20	4.423	3.861	0.686	3.153	10.376
DNN	30	4.906	4.326	1.204	3.216	11.710
DNN	40	7.808	6.989	2.350	5.992	16.634
DNN	50	5.744	5.164	1.085	3.958	13.998
DNN	60	6.659	5.001	1.762	5.594	14.302
DNN	70	10.580	7.243	2.361	9.530	19.710
DNN	80	11.513	8.200	4.135	9.519	24.350
DNN	90	15.036	9.960	4.628	12.797	27.569

The largest errors are likewise consistent with the geometry of the site rather than anomalous. The surveyed area spans 95.5 m × 24.2 m, so the largest geometrically possible error is its 98.6 m diagonal, and the median separation between two RPs is 29.3 m; the 34.4 m maximum is therefore a mid-range confusion rather than an extreme one, and it occurs in 3 of the 105 estimates, at two distinct locations. All three share one signature: the true RP was absent from the training partition, and the fingerprint was matched to a point displaced almost entirely along the building’s long axis (mean |Δx|=34.1 m against |Δy|=1.7 m). This is along-corridor RSSI aliasing, not a signal-quality problem, as those fingerprints detect 28 APs against a dataset median of 25.

The statistics further show that the near-zero mass is not a fixed property of kNN but tracks how much of the test set the radiomap still covers. It is amplified by redundancy in the raw data: 56% of the fingerprints duplicate another fingerprint’s RSSI vector exactly, so at 0% omission 57 of the 105 test points have a training neighbor at distance zero. The number of test fingerprints whose RP remains in the training set falls from 76 at 0% omission to 72, 68, 55 and 44 at 10%, 20%, 30% and 50% respectively, and the count of numerically zero kNN errors (below $10^{-9}$ m) follows it closely at 66, 62, 58, 48 and 38; by 90% omission only 3 remain. At 60% and 70% omission the tuned kNN instead selects seven distance-weighted neighbors, which averages several RP labels and removes most exact zeros. XGBoost produces no error below $10^{-9}$ m at any omission level. The right-skew reported above is therefore strongest exactly where the radiomap is densest, and it relaxes as sparsification removes the RPs that made exact retrieval possible.

## Appendix C. Evaluation of Quality Filtering of Synthetic Fingerprints

We evaluated quality filtering of synthetic fingerprints introduced in Section 3.3.3. We generated synthetic fingerprints using GAN and VAE, then evaluated their accuracy with and without quality filters. Because a single stochastic run can hide a small effect, we repeated experiments for each (omission level, generator) pair over 10 random seeds, then averaged. Table A3 reports the mean MEE (m) ± standard deviation under each stage of the quality-filtering pipeline (*none*, *clip*, *clip+mask*, *full*). The rightmost column gives the paired full–none difference (negative indicates filtering helps). In nine of the ten cases, the full filtering brings accuracy gains ranging from 0.03 m to 0.63 m. Only in one case, accuracy worsened by 0.21 m. We conclude that the quality filter is weakly but consistently beneficial, most useful at high sparsity, and never meaningfully harmful.

**Table A3:** Localization MEE accuracy (m) with and without quality filters.

Omit %	Gen	None	Clip	Clip + Mask	Full	Full–None
30	VAE	4.20 ± 0.09	4.19 ± 0.10	4.22 ± 0.22	4.17 ± 0.24	−0.03
30	GAN	4.25 ± 0.17	4.25 ± 0.17	4.13 ± 0.20	4.21 ± 0.18	−0.04
50	VAE	4.48 ± 0.40	4.46 ± 0.39	4.19 ± 0.24	4.32 ± 0.34	−0.16
50	GAN	4.33 ± 0.33	4.33 ± 0.33	4.37 ± 0.19	4.20 ± 0.27	−0.13
70	VAE	7.37 ± 0.66	7.34 ± 0.59	7.40 ± 0.33	7.30 ± 0.43	−0.07
70	GAN	7.24 ± 0.34	7.24 ± 0.34	7.40 ± 0.43	7.14 ± 0.31	−0.10
80	VAE	7.76 ± 0.38	7.72 ± 0.40	7.56 ± 0.22	7.62 ± 0.17	−0.14
80	GAN	7.42 ± 0.45	7.42 ± 0.45	7.76 ± 0.27	7.63 ± 0.29	+0.21
90	VAE	9.98 ± 1.04	10.04 ± 1.02	9.10 ± 0.36	9.35 ± 0.47	−0.63
90	GAN	9.52 ± 0.70	9.52 ± 0.70	9.20 ± 0.30	9.31 ± 0.65	−0.21

## References

[1] He S., Chan S.H.G. (2016). Wi-Fi fingerprint-based indoor positioning: Recent advances and comparisons. IEEE Commun. Surv. Tutor..

[2] Khalajmehrabadi A., Krishnamurthy N., Akopian D. (2017). Modern WLAN fingerprinting indoor positioning methods and deployment challenges. IEEE Commun. Surv. Tutor..

[3] Saeed M.T.M., Yousif M.A.A., Ozturk I. (2025). Mitigating device heterogeneity for enhanced indoor positioning system performance using deep feature learning. IEEE Access.

[4] Ali H.A.H., Seytnazarov S. (2023). Human walking direction detection using wireless signals, machine and deep learning algorithms. Sensors.

[5] Kingma D.P., Welling M. (2013). Auto-encoding variational Bayes. arXiv.

[6] Yoon N., Jung W., Kim H. (2024). DeepRSSI: Generative Model for Fingerprint-Based Localization. IEEE Access.

[7] Njima W., Chafii M., Chorti A., Shubair R.M., Poor H.V. (2021). Indoor localization using data augmentation via selective generative adversarial networks. IEEE Access.

[8] Nabati M., Navidan H., Shahbazian R., Ghorashi S.A., Windridge D. (2020). Using synthetic data to enhance the accuracy of fingerprint-based localization: A deep learning approach. IEEE Sens. Lett..

[9] Chan Y., Lin P.Y., Tseng Y.Y., Chen J.J., Tseng Y.C. Learning-Based WiFi Fingerprint Inpainting via Generative Adversarial Networks. Proceedings of the IEEE International Conference on Computer Communications and Networks (ICCCN).

[10] Ho J., Jain A., Abbeel P. Denoising diffusion probabilistic models. Proceedings of the Advances in Neural Information Processing Systems (NeurIPS).

[11] Yang L., Finnerty P., Ohta C. Diffusion Model-based RSSI Fingerprint Generation for Indoor Localization in Dynamic Environments. Proceedings of the IEEE Wireless and Optical Communications Conference (WOCC).

[12] Zhumangali I., Seytnazarov S. Synthetic Wi-Fi Fingerprint Generation Using a Diffusion Probabilistic Model. Proceedings of the IEEE Vehicular Technology Conference (VTC2025-Fall).

[13] Ali H.A.H., Seytnazarov S. Detecting human walking direction using Wi-Fi signals. Proceedings of the International Conference on Applied Soft Computing and Communication Networks.

[14] Barnwal S., Peng W. Crowdsensing-based WiFi Indoor Localization using Feed-forward Multilayer Perceptron Regressor. Proceedings of the International Conference on Computational Intelligence in Data Science (ICCIDS).

[15] Esmaeili Gorjan H., Gil Jiménez V.P. (2024). Improving indoor Wi-Fi localization by using machine learning techniques. Sensors.

[16] Bahl P., Padmanabhan V.N. RADAR: An in-building RF-based user location and tracking system. Proceedings of the IEEE Information Communications (INFOCOM).

[17] Drucker H., Burges C.J.C., Kaufman L., Smola A., Vapnik V. Support vector regression machines. Proceedings of the Advances in Neural Information Processing Systems (NeurIPS).

[18] Chen T., Guestrin C. XGBoost: A scalable tree boosting system. Proceedings of the ACM International Conference on Knowledge Discovery and Data Mining (SIGKDD).

[19] Song S., Lee J., Park M., Lee S. CNNLoc: Deep-Learning Based Indoor Localization with Wi-Fi Fingerprinting. Proceedings of the IEEE International Conference on Indoor Positioning and Indoor Navigation (IPIN).

[20] Neupane I., Shahrestani S., Ruan C. (2024). Indoor Localization of Resource-Constrained IoT Devices Using Wi-Fi Fingerprinting and Convolutional Neural Network. Proceedings of the Australasian Computer Science Week.

[21] Hoang M.T., Yuen B., Dong X., Lu T., Westendorp R., Reddy K. (2019). Recurrent neural networks for accurate RSSI indoor localization. IEEE Internet Things.

[22] Goodfellow I., Pouget-Abadie J., Mirza M., Xu B., Warde-Farley D., Ozair S., Courville A., Bengio Y. Generative adversarial nets. Proceedings of the Advances in Neural Information Processing Systems (NeurIPS).

[23] Mumuni A., Mumuni F. (2022). Data augmentation: A comprehensive survey of modern approaches. Array.

[24] Chen X., Li H., Zhou C., Liu X., Wu D., Dudek G. (2022). Fidora: Robust WiFi-based indoor localization via unsupervised domain adaptation. IEEE Internet Things J..

[25] Chidlovskii B., Antsfeld L. Semi-Supervised Variational Autoencoder for WiFi Indoor Localization. Proceedings of the IEEE International Conference on Indoor Positioning and Indoor Navigation (IPIN).

[26] Wu S., Zeng X., Zhang M., Cumanan K., Waraiet A., Chu Z., Xu K. (2025). LCVAE-CNN: Indoor Wi-Fi fingerprinting CNN positioning method based on LCVAE. IEEE Internet Things.

[27] Kim D., Park J.H., Suh Y.J. (2025). A Wi-Fi Fingerprinting Indoor Localization Framework Using Feature-Level Augmentation via Variational Graph Auto-Encoder. Electronics.

[28] Njima W., Bazzi A., Chafii M. (2022). DNN-Based Indoor Localization Under Limited Dataset Using GANs and Semi-Supervised Learning. IEEE Access.

[29] Yoo J. (2024). Wi-Fi fingerprint indoor localization by semi-supervised generative adversarial network. IEEE Sens..

[30] Junoh S.A., Pyun J.Y. (2023). Enhancing indoor localization with semi-crowdsourced fingerprinting and GAN-based data augmentation. IEEE Internet Things.

[31] Guo Z., Zhang X., Lu Y. Improve indoor localization accuracy by enriching CSI fingerprints using conditional diffusion probabilistic model. Proceedings of the IEEE International Conference on Communications (ICC).

[32] Zang R., Yan D., Wang B., Yan Y. Wi-Fi Based Indoor Human Trajectory Tracking with Diffusion-Model. Proceedings of the IEEE International Conference on Parallel and Distributed Systems (ICPADS).

[33] Liu Z., Zhang S., Liu Q., Zhang H., Song L. (2025). Wifi-diffusion: Achieving fine-grained wifi radio map estimation with ultra-low sampling rate by diffusion models. IEEE J. Sel. Areas Commun..

[34] Peebles W., Xie S. Scalable diffusion models with transformers. Proceedings of the IEEE International Conference on Computer Vision (ICCV).

[35] Yan Q., Dai W., Xiong W., Wang H.M. Diffusion model-based fingerprint synthesis for indoor localization. Proceedings of the SPIE International Conference on Artificial Intelligence and Pattern Recognition (AIPR).

[36] Shao S., Lu H., Luo D., Lu T., Dong X. (2018). UTSIndoorLoc: A WLAN fingerprint-based indoor localization dataset. Data Brief..

[37] Hoang M.T., Dong X., Lu T. (2019). Wi-Fi RSSI Indoor Localization.

[38] Moenning D., Dodgson N.A. (2003). Fast Marching Farthest Point Sampling.

[39] Akiba T., Sano S., Yanase T., Ohta T., Koyama M. Optuna: A next-generation hyperparameter optimization framework. Proceedings of the ACM International Conference on Knowledge Discovery and Data Mining (SIGKDD).

[40] Optuna: A Hyperparameter Optimization Framework. https://github.com/optuna/optuna.

[41] Malikov N. Wi-Fi Localization GitHub Repository. https://github.com/m-nurbek/wifi_localization.

[42] Chen T., Zhang R., Hinton G. Analog bits: Generating discrete data using diffusion models with self-conditioning. Proceedings of the International Conference on Learning Representations (ICLR).

[43] Nichol A., Dhariwal P. Improved denoising diffusion probabilistic models. Proceedings of the International Conference on Learning Representations (ICLR).

[44] Torres-Sospedra J., Montoliu R., Martínez-Usó A., Avariento J.P., Arnau T.J., Benedito-Bordonau M., Huerta J. UJIIndoorLoc: A New Multi-building and Multi-floor Database for WLAN Fingerprint-based Indoor Localization Problems. Proceedings of the 2014 International Conference on Indoor Positioning and Indoor Navigation (IPIN).

